# Unraveling new therapeutic targets in ankylosing spondylitis: Multi-omics Mendelian randomization on immune cells, metabolites, and inflammation proteins

**DOI:** 10.1097/MD.0000000000042177

**Published:** 2025-04-18

**Authors:** Kai Du, Chen-Yu Zhang, Ao Li, Qi-Heng Zuo, Ren Guo, Shu-Ming Li

**Affiliations:** a Beijing University of Chinese Medicine, Beijing, People’s Republic of China; b Department of Pain Medicine, Beijing Hospital of Traditional Chinese Medicine, Capital Medical University, Beijing, China.

**Keywords:** ankylosing spondylitis, genetic epidemiology, immune cells, inflammation proteins, metabolites, multi-omics Mendelian randomization

## Abstract

This study aims to identify novel immunological, metabolic, and inflammatory determinants of ankylosing spondylitis (AS) using Mendelian randomization (MR), offering new insights into its pathogenesis and potential therapeutic interventions. Employing a bidirectional, secondary validation two-sample MR, this study investigated causal associations among 1400 serum metabolites, 731 immune cell traits, and 91 circulating inflammatory proteins with AS. Instrumental variables were identified using PLINK for minimal linkage disequilibrium, applying strict significance thresholds. Various MR methodologies, including inverse variance weighted, weighted median, and MR Egger, were applied to validate causal links. Sensitivity analyses, incorporating heterogeneity and pleiotropy tests, were performed to evaluate the robustness of the results. The false discovery rate correction was applied to adjust for multiple comparisons, while the MR Steiger directionality test and bidirectional MR analysis validated the causation direction. Secondary validation with data from diverse sources was undertaken to confirm the reliability of the findings. After false discovery rate correction, associations were identified between AS etiology and 9 immune cell traits, 2 serum metabolites, and 2 inflammatory proteins. Notably, the presence of CX3CR1 on monocytes and the absolute count of CD62L- CD86+ myeloid dendritic cells (DCs) were associated with an increased risk of AS. In contrast, expression of HLA DR on DCs, including myeloid and plasmacytoid DCs, and on CD14- CD16- monocytes, along with CD64 expression across various monocyte subsets (monocytes, CD14+ CD16+, and CD14+ CD16-), correlated with a decreased risk of AS development. Serum metabolites, specifically levels of hexadecanedioate and Bilirubin (E, Z or Z, E), were also linked to a reduced risk of AS. Regarding inflammatory factors, interleukin-6 levels were inversely associated with AS morbidity, whereas TNF-beta levels were positively correlated with higher AS morbidity. Neither bidirectional MR nor MR Steiger tests provided evidence supporting reverse causation. This study sheds light on the complex interactions between immune cells traits, metabolites, and inflammatory proteins in AS, offering new insights into its pathophysiology. The findings underscore the importance of the immune–metabolic–inflammation network in AS, suggesting novel biomarkers for diagnosis and targets for therapy.

## 1. Introduction

Ankylosing spondylitis (AS) is a chronic, progressive rheumatic condition predominantly affecting the spine and pelvis, with potential extension to other joints and organs. This disease is marked by persistent low back pain, morning stiffness, and fatigue, significantly impairing patients’ mobility, work capacity, and social interactions. Epidemiologically, AS manifests a global incidence of approximately 6.1 per 100,000 individuals annually, displaying a gender disparity with males experiencing roughly twice the prevalence of females across various populations, albeit with noted exceptions.^[1,2]^ Regionally, prevalence rates vary, from 0.20% to 0.25% in North America and Europe, to higher incidences of 0.29% among military populations in mainland China and up to 0.35% in Northern Arctic communities. This variation is largely attributed to the differential prevalence of the HLA-B27 antigen, a known genetic risk factor for AS. However, the presence of HLA-B27 alone does not account for all AS cases, underscoring the role of additional genetic and environmental factors in disease development.^[3–6]^

While the associations with genetics, immune responses, and environmental factors are recognized, the precise causative mechanisms of AS are yet to be fully elucidated. This underscores the importance of integrating multi-omics approaches to uncover the complex interplay of these factors. Traditionally focused on T helper (Th)17 cells and their cytokines, interleukin (IL)-17 and IL-23, AS immunopathology has evolved to include a broader spectrum of immune cells and cytokines, reflecting the disease’s complex immune regulation.^[7,8]^ Advances in immunology have emphasized the roles of regulatory T cells (Treg), natural killer cells, and mesenchymal stem cells. Additionally, the involvement of IL-22-producing cells, gamma delta T cells, and regulatory B cells has been recognized, shedding new light on AS’s immune dynamics and unveiling potential therapeutic targets.^[9–15]^

Complementing this immunological perspective, recent metabolomic studies have shed light on the intricate interplay between metabolism and immune function in AS. Alterations in lipid profiles, particularly ceramides and sphingolipids, have been implicated in the inflammation characteristic of AS.^[16]^ Additionally, the gut microbiome’s influence, through metabolites like short-chain fatty acids, suggests a potential gut–joint axis in the disease’s pathology.^[17]^ This axis is further complicated by the involvement of microbial metabolites like 3-hydroxypropionic acid, which could contribute to neoantigen formation in AS through cysteine carboxyethylation. Moreover, perturbations in purine and tryptophan pathways suggest underlying oxidative stress and immune dysfunction, offering new insights into AS pathogenesis.^[18]^

In the realm of circulating inflammatory proteins, recent studies have moved beyond the traditional focus on Th17 cell-associated cytokines like IL-17 and IL-23, exploring the roles of chemokines such as C–X–C motif chemokine 5 and C–C motif chemokines. These chemokines are now understood to be instrumental in AS inflammation, influencing leukocyte movement and impacting inflammatory responses through the regulation of target cell adhesion molecules and cytoskeletal proteins. This evolving landscape of inflammatory mediators, including various interleukin family members, underscores a more complex and nuanced immunoregulatory network in AS.

The pathogenesis of AS intricately intertwines immune responses, metabolic pathways, and inflammatory proteins. Immune cells and metabolic states are closely linked, where processes like glycolysis and fatty acid oxidation directly influence immune cell behavior. Metabolites, such as ceramides, act as immunomodulators, shaping the immune landscape. Simultaneously, cytokines and chemokines bridge immune and metabolic processes, affecting lipid metabolism and insulin sensitivity. This interplay creates a feedback loop where inflammation drives metabolic changes, further influencing immune dysregulation. In AS, this is evident as specific immune cells adopt distinct metabolic pathways, with alterations in metabolites exacerbating inflammatory responses, thereby perpetuating disease progression. Such insights into the immune–metabolic–inflammation network offer a novel perspective on AS’s underlying mechanisms, emphasizing the need for a multifaceted exploration of its varied manifestations and impact.

In exploring the multifaceted etiology of AS, our research leverages the Mendelian randomization (MR) approach. MR, recognized for its methodological rigor, uses genetic variants as instrumental variables (IVs) to draw causal connections between modifiable risk factors: namely, immune cell functionality, metabolite profiles, and inflammatory protein expression, and AS. Its design, which parallels the random allocation of alleles akin to clinical trial randomization, markedly reduces confounding factors and addresses reverse causation concerns, enabling more precise causal inferences than traditional observational studies. This investigation aims to shed light on novel immunological, metabolic, and inflammatory determinants within the intricate immune–metabolic–inflammation network, deciphering their roles in AS’s pathogenesis and progression. Through identifying causal links between genetic variants and AS risk factors, we aspire to discover biomarkers for disease susceptibility, progression, and treatment efficacy. Additionally, our analysis seeks to understand how these elements jointly influence AS pathology, pinpointing potential therapeutic targets. The ultimate goal is to lay a solid groundwork for the innovation of diagnostic tools and personalized treatment plans for AS, thus informing clinical practice and improving patient care outcomes.

## 2. Materials and methods

### 2.1. Study design and basic assumptions of MR

**Figure 1.**
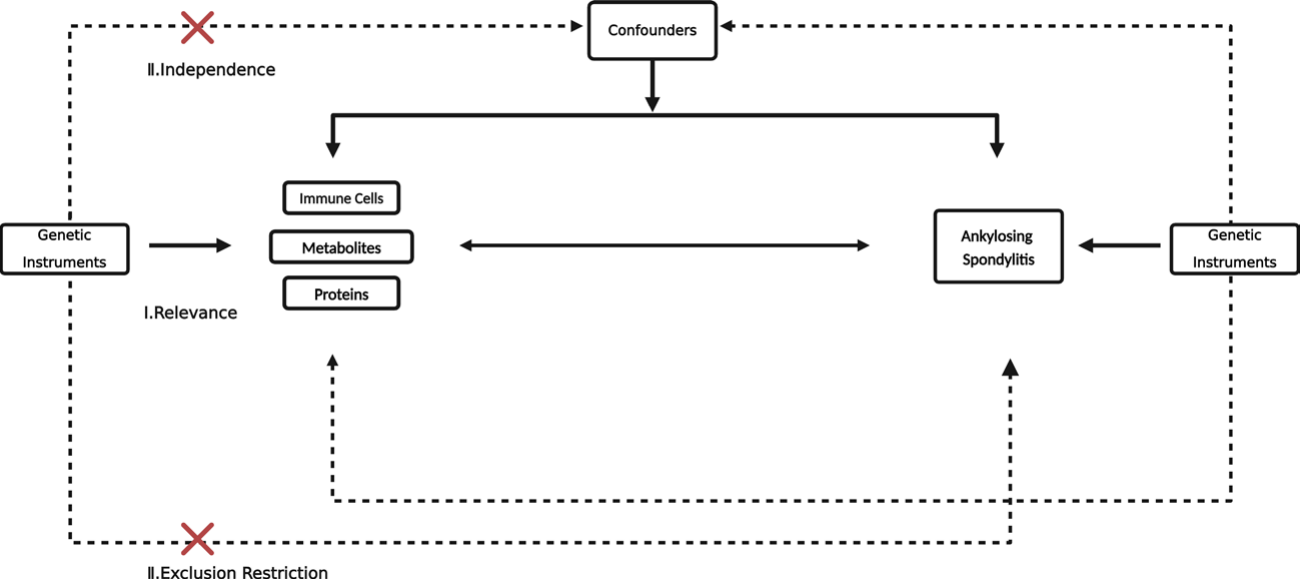
Bidirectional Mendelian randomization design.

Using a summary-level dataset of genome-wide association study (GWAS), we assessed causal associations between 731 immune cell traits (7 groups), 1400 serum metabolites, 91 inflammation proteins, and risk of AS with the bidirectional, secondary validation two-sample MR (T). MR is based on 3 assumptions crucial for our study’s integrity: Assumption Ⅰ ensures that the IVs we selected are strongly linked to the exposure (immune cells traits, metabolites, inflammation proteins), guaranteeing their effectiveness in MR analysis. Assumption Ⅱ confirms that our IVs are not associated with confounders, enhancing the credibility of our causal inferences. Assumption Ⅲ establishes that our IVs affect AS risk solely through their impact on the exposures, preventing bias from alternative pathways. This study adheres to the guidelines provided by the “Strengthening the Reporting of Observational Studies in Epidemiology using Mendelian Randomization” (STROBE-MR) checklist. Figure 1 illustrates the 3 assumptions of MR and the study design.

### 2.2. Data sources and populations

In our investigation, we utilized the R10 release of the FinnGen database, a rich and diverse genetic repository, to obtain the GWAS summary statistics for AS. This dataset, identified as finngen_R10_M13_ANKYLOSPON_4, is notable for its comprehensive collection of genetic data drawn from a wide-ranging European population. The dataset encompasses 3162 cases diagnosed with AS and 294,770 control subjects, nearly covering 21,303,167 SNPs. To mitigate population stratification, rigorous principal components analysis was applied, enhancing the validity of genetic findings by ensuring any associations identified are reflective of true biological links, rather than artifacts of population structure. Downloading from the GWAS Catalog (accession numbers GCST0001391–GCST0002121), we delved into a comprehensive analysis of 731 distinct immunophenotypes.^[19]^ This included 118 absolute cell counts, 389 median fluorescence intensities, as well as 32 morphological parameters, and 192 relative cell counts. The original GWAS data analyses employed linear mixed models and multigenerational family data to address population stratification, enhancing the genetic insights into immune traits. Supported by high-quality genotyping, imputation, and adjustments for age and sex, these methods ensured the association findings’ reliability, effectively mitigating potential biases. In the analysis of 1091 blood metabolites and 309 metabolite ratios using data from the Canadian Longitudinal Study of Aging cohort, the original researchers meticulously processed data from 8299 individuals (accession numbers GCST90199621–GCST90201020). This included the classification of 850 plasma metabolites into 8 principal pathways: lipid, amino acid, xenobiotics, nucleotide, cofactor and vitamins, carbohydrate, peptide, and energy, with the remainder being less distinctly characterized. The original study team’s careful selection of unrelated individuals of European descent and their rigorous genotype quality control measures, including adjustments for the first 10 genetic principal components, effectively addressed the issue of population stratification. These methodological choices ensured the integrity and reliability of the genetic associations with the metabolites and metabolite ratios identified in their comprehensive genetic analyses.^[20]^ Ninety-one circulating inflammatory proteins were measured in a cohort of 14,824 participants using the Olink Target platform, with GWAS summary statistics available from the EBI GWAS Catalog (accession numbers GCST90274758–GCST90274848). The study addressed population stratification by incorporating genetic principal components as covariates in GWAS analyses, thus adjusting for ancestry differences. This approach, coupled with the careful selection of participants and standardized genotyping, along with a thorough meta-analysis and validation, effectively minimized the influence of population stratification, ensuring the reliability of the identified pQTL associations.^[21]^

### 2.3. Selection of the genetic IVs

In our methodology for identifying IVs, we adopted a dual approach to optimize both SNP selection and linkage disequilibrium management. SNPs were initially chosen based on a *P*-value threshold of 1 × 10^-5^, a level designed to encompass a comprehensive range of genetic variations relevant to AS’s complex immunological aspects. This threshold ensures the inclusivity of significant variants, balancing specificity with the need to capture subtle yet important genetic effects. For linkage disequilibrium management, we employed the PLINK clumping procedure with an *r*² threshold of 0.1 over a 500 kb range. This *r*² threshold was strategically selected to include SNPs with moderate correlations, thereby ensuring a robust and reliable dataset. The 500 kb range effectively minimizes potential confounding from overlapping genetic signals. This methodological rigor is crucial in accurately dissecting the intricate genetic landscape of AS, allowing us to explore the nuanced genetic interplays characteristic of this complex disease. This set of criteria is consistent with the original data sources and the methods used in previous studies, ensuring the continuity and relevance of our research within the broader scientific context.^[22–24]^ In the reverse MR analysis within our study, the screening criteria were carefully defined: we set the *P*-value threshold below 5 × 10^-8^, adhered to an *r*² threshold of 0.001, and maintained a kb range of 10,000. This approach further exemplifies our commitment to advancing our understanding of AS, building on a foundation of established scientific rigor. To ascertain the potency of our chosen IVs, we determined the proportion of phenotypic variation explained (PVE) for each IV, as well as the corresponding F statistic. *R*² is the total proportion of PVE by SNPs in the database, which is calculated by [2 × EAF × (1 − EAF) × beta^2^]/(SE² × N), where EAF represents the effect allele frequency, N is the sample size, beta is the allele effect value, and SE is the stand error. The F statistic was calculated as F = (PVE ×  (N − 2))/(1 − PVE), IVs with small F statistics (F < 10) were regarded as weak variables.

### 2.4. Statistical analysis and sensitivity analysis

To elucidate the putative causal relationships between immune cells traits, metabolites, and AS, we embarked on a comprehensive analysis using the TwoSampleMR R package (version 0.5.6). We deployed a suite of MR techniques, encompassing MR Egger, weighted median, inverse variance weighted (IVW), simple mode, and weighted mode. We performed a series of sensitivity analyses to ensure the reliability of the outcomes. Heterogeneity, defined as variations in causal estimates across different IVs, can obscure true causal relationships in MR studies. Upon detecting significant heterogeneity, which could potentially undermine the validity of our causal inferences, we employed the multiplicative random effects (MRE) model within the IVW analysis to adjust for these variations, thereby enhancing the robustness of our results. Addressing horizontal pleiotropy, wherein IVs may influence the outcome through non-exposure-related pathways, constituted a pivotal facet of our analysis. Through MR Egger regression, we conducted a quantitative assessment and correction for horizontal pleiotropy, underpinning the robustness of our causal estimates. The MR-PRESSO test played a crucial role by accurately identifying and eliminating pleiotropic outliers, thus refining our set of IVs to bolster accuracy. This was further buttressed by the weighted median method, offering resilience against pleiotropy by delivering reliable causal estimates even when a significant proportion of the IVs were invalid. Rigorous application of the leave-one-out approach, corroborated the stability and reliability of our causal inferences, ensuring that no individual IV disproportionately influenced our outcomes. Visual diagnostics, encompassing scatter, funnel, and forest plots, provided unequivocal, empirical substantiation of our results’ resilience against pleiotropy, illustrating the meticulous and systematic approach we employed to mitigate bias and affirm the integrity of our findings.^[25]^ To further ensure the accuracy of our causal direction and to guard against the pitfalls of reverse causality, we implemented the MR Steiger directionality test along with bidirectional MR analysis. The MR Steiger test is instrumental in verifying that our IVs influence the outcome through the intended exposure pathway. Conversely, the bidirectional MR analysis provides a comprehensive examination of causality in both directions by interchanging the roles of exposure and outcome. Through this comprehensive and meticulous sensitivity analysis, we have taken extensive measures to minimize bias, thereby affirming the validity and integrity of our conclusions within the complex etiology of diseases.

To enhance the integrity of our statistical analysis amidst the complexity of multiple comparisons, we incorporated the false discovery rate (FDR) correction employing the Benjamini-Hochberg procedure for *P*-value adjustment. This methodological choice was pivotal in ensuring the resilience of our findings against potential false positives, an inherent risk in analyses spanning extensive datasets. The selection of a 0.05 FDR threshold was deliberate, reflecting a standard practice in genomic studies that judiciously balances the dual imperatives of minimizing false discoveries while fostering the identification of genuine associations. This threshold was carefully chosen to underpin the conservative yet insightful delineation of statistically significant relationships. Such relationships are signified by odds ratios (OR) accompanied by 95% confidence intervals (CI), where an OR >1, alongside a 95% CI that does not straddle 1, and a *P*-value below the FDR-adjusted threshold, denotes a statistically meaningful association. The OR represents the likelihood of the occurrence of AS given the presence of specific biomarkers, thus serving as a cornerstone for inferring causal relationships within our study.^[26]^

## 3. Results

Our bidirectional MR analysis explored causal links between 731 immune cell traits, 1400 serum metabolites, 91 inflammatory proteins, and AS. A summarized overview of all significant findings across these domains, including 9 immune cell traits, 2 serum metabolites, and 2 inflammatory proteins with notable causal links after FDR correction, is presented in Table S1, Supplemental Digital Content, https://links.lww.com/MD/O714.

### 3.1. Bidirectional causality between immune cell traits and AS

**Figure 2.**
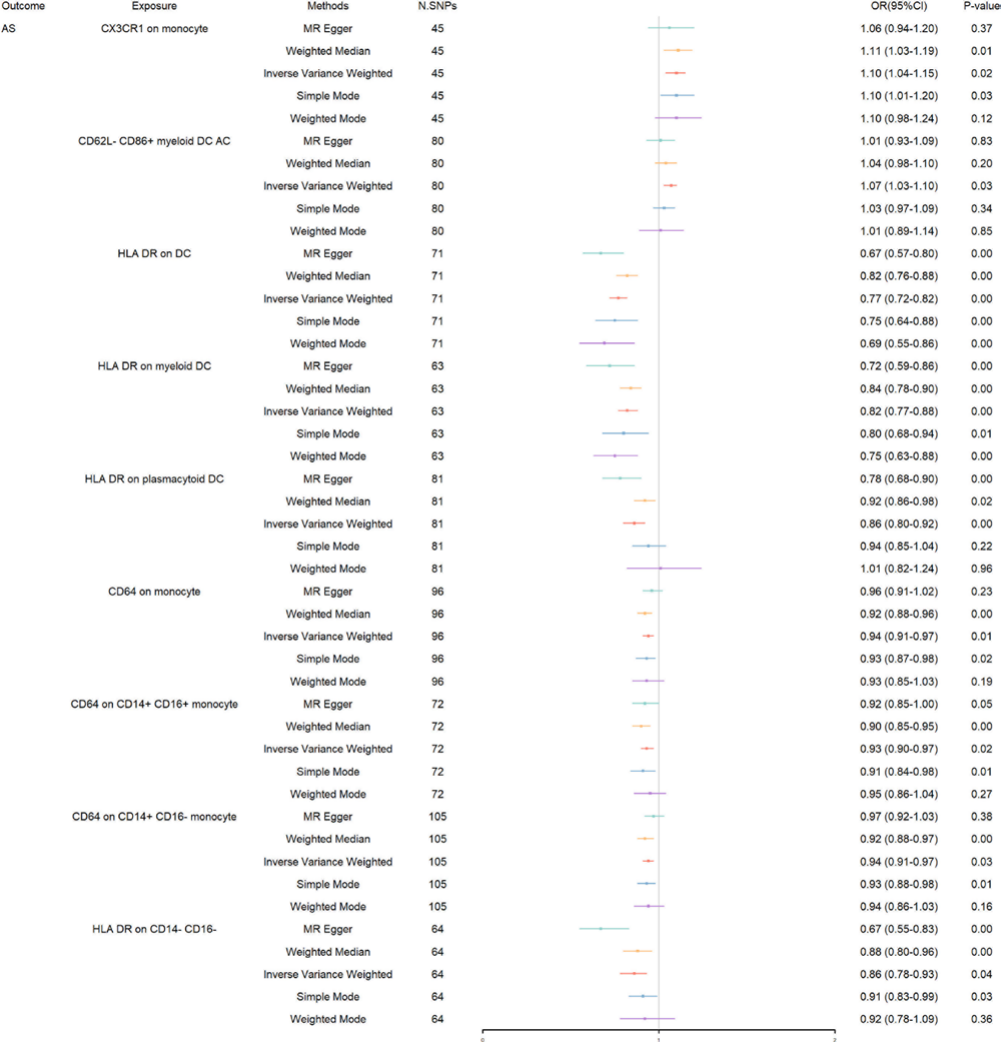
Bidirectional MR results in forest plot between immune cells and AS. AS = ankylosing spondylitis, MR = Mendelian randomization.

Our investigation into the genetic determinants of AS through MR analysis reveals significant insights into how specific immune cell traits influence the disease’s risk (Fig. 2). Starting with CX3CR1 expression in monocytes, we found a notable association with increased AS risk [IVW: OR 1.10, 95% CI 1.04–1.15, *P* = .024], a finding that gains further support from weighted median [OR 1.11, *P* = .007] and weighted mode [OR 1.10, *P* = .027], emphasizing its potential role in AS pathogenesis. Transitioning to the dendritic cell (DC) compartment, CD62L- CD86+ myeloid DC absolute count also showed an elevated risk association [IVW: OR 1.07, 95% CI 1.03–1.10, *P* = .027], albeit with less consistency across other MR methods, suggesting a nuanced contribution to AS susceptibility. The narrative takes a compelling turn with HLA DR expression across different DC types, consistently illustrating a protective effect against AS. Specifically, HLA DR on DC [IVW: OR 0.77, 95% CI 0.72–0.82, *P* < .001], on myeloid DC [IVW: OR 0.82, 95% CI 0.77–0.88, *P* < .001], and on plasmacytoid DC [IVW: OR 0.86, 95% CI 0.80–0.92, *P* = .003] all showed significant protective associations, strongly supported by weighted median and weighted mode analyses. These results not only underscore HLA DR’s critical immunoregulatory role but also highlight its potential as a key modulator of AS risk. Further exploring the landscape of immune modulation, the CD64 expression on various monocyte subsets reveals a protective landscape across its variants, with monocytes [IVW: OR 0.94, 95% CI 0.91–0.97, *P* = .007], CD14 + CD16 + monocytes [IVW: OR 0.93, 95% CI 0.90–0.97, *P* = .024], and CD14 + CD16- monocytes [IVW: OR 0.94, 95% CI 0.91–0.97, *P* = .029] all demonstrating significant protective effects. This pattern, echoed by findings in weighted median and weighted mode, suggests CD64’s involvement in mitigating AS risk, further delineating the complex interplay of immune cells traits in AS. Lastly, HLA DR expression on CD14- CD16- cells fortifies the protective narrative against AS [IVW: OR 0.86, 95% CI 0.78–0.93, *P* = .041], with corroborative evidence from weighted median [OR 0.88, *P* = .004] and weighted mode [OR 0.91, *P* = .025], reinforcing the overarching theme of HLA DR’s broad immunomodulatory impact in reducing AS risk.

The F-statistic values, robust across the board [ranging from 35.414–54.572], alongside *R*² metrics [spanning 0.11–0.29], underscore the IVs’ strength and the analyses’ explanatory power. To manage heterogeneity in our analysis, specifically within groups including HLA DR on DC, HLA DR on myeloid DC, HLA DR on plasmacytoid DC, CD64 on CD14+ CD16- monocyte, and HLA DR on CD14- CD16-, we employed the IVW-MRE model, thereby enhancing the reliability of our results. Encountering pleiotropy within the HLA DR on CD14- CD16- subgroup, we applied the MR-PRESSO method for a refined correction. However, the persistence of pleiotropy, as evidenced by a post-correction Global Test P of 0.001, underscores the complexity of these genetic interactions and mandates a cautious interpretation of the observed associations.

The Steiger test did not reveal any evidence of reverse causality between immune cell traits and AS, providing additional confidence in the directionality of the reported associations. To further substantiate our conclusions and explore the possibility of inverse relationships, we endeavored to conduct bidirectional MR analyses. Despite broadening our SNP selection criteria: lowering the *P*-value threshold to 1 × 10^-5^, accepting an *R*² of 500, and expanding the distance to 0.1 kb, we faced challenges in identifying suitable IVs for these reverse analyses. This difficulty highlights the complexities and limitations in sourcing genetic instruments for reverse causation scenarios, especially in the context of multifaceted diseases like AS. All detailed results of our MR analyses, including other MR methods and sensitivity analyses, are provided in Table S2, Supplemental Digital Content, https://links.lww.com/MD/O714 for a comprehensive overview. Figures S1 to S36, Supplemental Digital Content, https://links.lww.com/MD/O715 display scatter plots, funnel plots, leave-one-out analyses, and forest plots, visualizing the results.

### 3.2. Bidirectional causality between metabolites and AS

**Figure 3.**
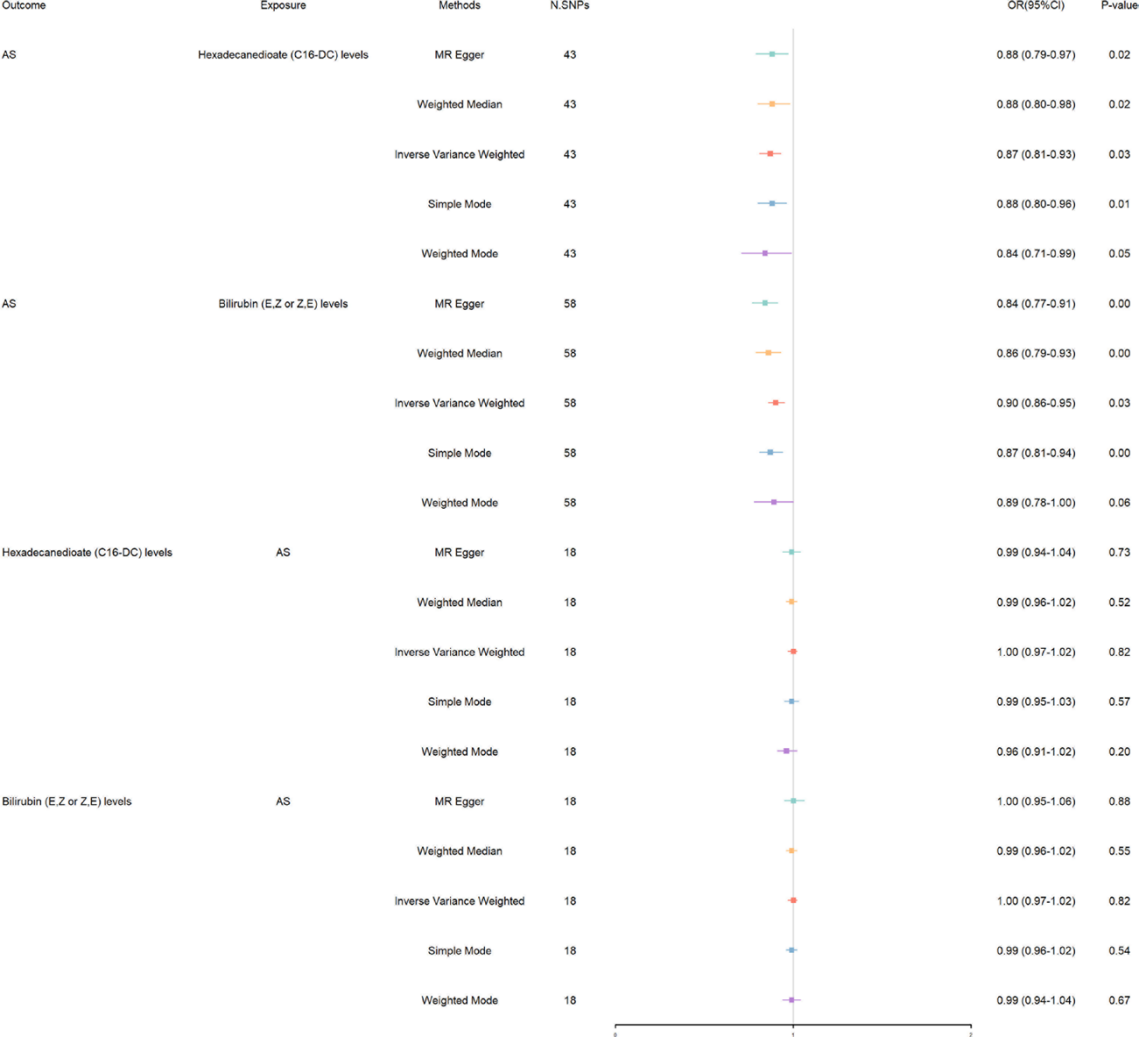
Bidirectional MR results in forest plot between serum metabolites and AS. AS = ankylosing spondylitis, MR = Mendelian randomization.

Our investigation extends to the metabolic domain, examining how hexadecanedioate (C16-DC) and bilirubin (E, Z or Z, E) levels influence AS risk (Fig. 3). For hexadecanedioate levels, a consistent pattern emerged across multiple MR methods, suggesting a protective effect against AS. The IVW method highlighted this association [IVW: OR 0.87, 95% CI 0.81–0.93, *P* = .027], with supportive evidence from weighted median [OR 0.88, *P* = .018] and weighted mode [OR 0.88, *P* = .009], reinforcing the metabolic link to AS risk mitigation. Notably, the MR Egger analysis [OR 0.88, *P* = .017] further validated this protective trend, albeit with a threshold of statistical significance, suggesting a potential role of hexadecanedioate in reducing AS susceptibility. Turning our attention to Bilirubin (E, Z or Z, E) levels, the protective narrative against AS risk was even more pronounced. The IVW analysis [IVW: OR 0.90, 95% CI 0.86–0.95, *P* = .027] and weighted median [OR 0.86, *P* < .001] provided compelling evidence of Bilirubin’s protective role. This was robustly supported by weighted mode [OR 0.87, *P* = .001] and further explored in MR Egger [OR 0.84, *P* < .001]. These findings collectively suggest Bilirubin’s involvement in a protective metabolic pathway against AS.

Sensitivity analyses including *R*² and F-statistic values [hexadecanedioate: *R*² = 0.072, F = 58.671; Bilirubin (E, Z or Z, E) levels: *R*² = 0.142, F = 76.415] underscored the IVs’ strength and the analyses’ overall reliability. Furthermore, assessments of heterogeneity and pleiotropy revealed no significant concerns [hexadecanedioate: heterogeneity *P* = .894, pleiotropy *P* = .863; Bilirubin (E, Z or Z, E) levels: heterogeneity *P* = .496, pleiotropy *P* = .044, global test *P* = .529] confirming the stability of our findings (Table S3, Supplemental Digital Content, https://links.lww.com/MD/O714). Steiger tests and bidirectional MR analyses for C16-DC and Bilirubin (E, Z or Z, E) levels did not indicate reverse causation with AS risk, affirming the directionality of their protective effects on AS without evidence of reverse relationships. The results are visualized in Figures S37 to S44, Supplemental Digital Content, https://links.lww.com/MD/O715, which include scatter plots, funnel plots, analyses using leave-one-out, and forest plots.

### 3.3. Bidirectional causality between inflammation proteins and AS

**Figure 4.**
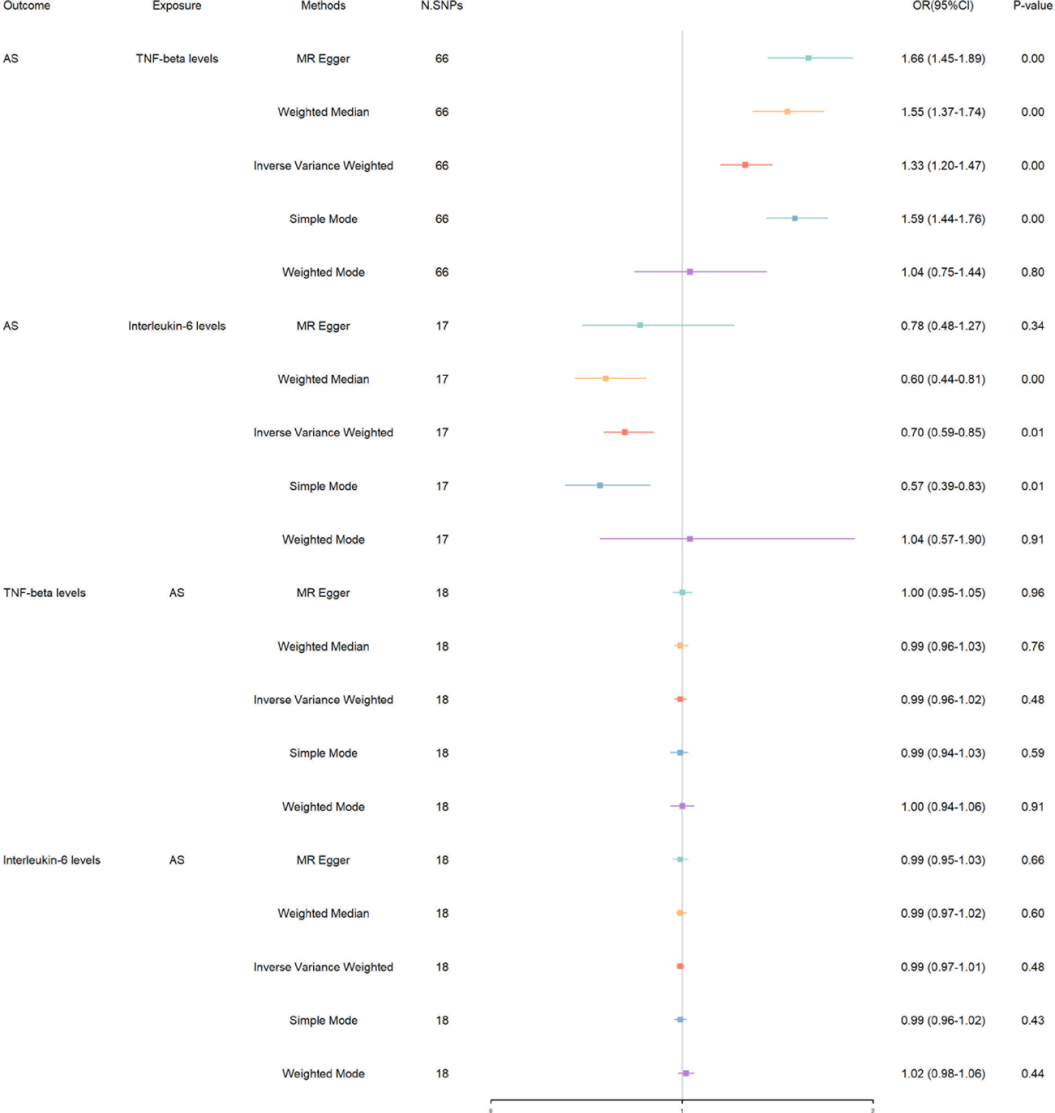
Bidirectional MR results in forest plot between inflammation proteins and AS. AS = ankylosing spondylitis, MR = Mendelian randomization.

The evidence robustly positions tumor necrosis factor-beta (TNF-β) levels as a significant enhancer of AS risk, with the IVW analysis presenting a clear association [IVW: OR 1.33, 95% CI 1.20–1.47, *P* < .001] (Fig. 4). This finding is consistently supported across weighted median [OR 1.55, *P* < .001] and weighted mode [OR 1.59, *P* < .001], underscoring TNF-β’s pro-inflammatory role in AS. However, the simple mode result [OR 1.04, *P* = .800] introduces a note of caution, suggesting the complexity of TNF-β’s impact on AS. On the other spectrum, IL-6 levels present a contrasting narrative, hinting at a protective effect against AS, albeit with a nuanced statistical landscape. The IVW method suggests a trend towards protection [IVW: OR 0.70, 95% CI 0.59–0.85, *P* = .009], a pattern echoed in weighted median [OR 0.60, *P* = .001] and weighted mode [OR 0.57, *P* = .010] analyses.

Sensitivity analyses, including [*R*² = 0.038, F = 58.794 for TNF-β] and [*R*² = 0.011, F = 33.324 for interleukin-6], underscore the strength of our IVs and the reliability of our findings. Upon applying MR-PRESSO, we observed significant heterogeneity and pleiotropy for TNF-β [heterogeneity *P* < .001, pleiotropy *P* < .001, global test *P* = .001], indicating the persistence of pleiotropic effects and complex interactions that influence AS risk. This complexity requires a cautious interpretation of TNF-β’s influence on AS. Conversely, IL-6 presented mild heterogeneity [*P* = .053] and nonsignificant pleiotropy [*P* = .619], suggesting a more direct association with AS. We addressed heterogeneity using the IVW-MRE model and managed pleiotropy with MR-PRESSO. Despite these measures, TNF-β’s residual pleiotropy highlights the necessity for deeper investigation into its role in AS. The Steiger test found no evidence of reverse causation for both markers, a conclusion supported by bidirectional MR analyses (Table S4, Supplemental Digital Content, https://links.lww.com/MD/O714). These findings solidify the directional influence of these markers on AS risk and underscore the importance of employing a multifaceted analytical approach to fully understand their roles in the pathogenesis of AS (Table S5, Supplemental Digital Content, https://links.lww.com/MD/O714). Visualization of the results is provided in Figures S44 to S52, Supplemental Digital Content, https://links.lww.com/MD/O715, featuring scatter plots, funnel plots, leave-one-out analyses, and forest plots.

### 3.4. Secondary validation with different sources of GWAS

To validate our initial findings regarding the genetic underpinnings of AS, we conducted secondary analyses using 3 genetic databases: ebi-a-GCST005529, ukb-a-88, and ukb-b-18194 (Table S6, Supplemental Digital Content, https://links.lww.com/MD/O714). The ebi-a-GCST005529 dataset, despite being enriched with AS-associated genetic markers, featured SNP counts below the robustness threshold necessary for validation, diminishing its analytical utility. Conversely, the ukb-a-88 and ukb-b-18194 datasets provided more substantial SNP counts but resulted in ORs and 95% CIs converging on 1.00, indicating no significant associations. This outcome could be attributed to demographic stratification, insufficient genetic variation among participants, or potential SNP selection biases, factors that underscore the challenges in dissecting the complex genetic etiology of multifactorial diseases like AS.

The absence of significant genetic associations in our secondary validation efforts underscores the intricate complexities and challenges in deciphering AS’s genetic landscape. These findings emphasize the necessity for future genetic studies to adopt more nuanced approaches, considering a broader spectrum of genetic variations and gene–environment interactions, to more accurately unravel the multifaceted genetic underpinnings of AS. Such comprehensive studies are essential to decode the complex genetic architecture of AS and to advance our understanding of its pathogenesis.

## 4. Discussion

Our study utilized MR to explore the complex relationships between immune cells traits, serum metabolites, and inflammatory factors about AS. We discovered that certain immune cell traits, specifically the presence of CX3CR1 on monocytes and CD62L- CD86+ myeloid DCs, are associated with an increased risk of AS. Conversely, the expression of HLA DR on various DC types, including myeloid, plasmacytoid, and the CD14- CD16- series, was found to decrease the risk of developing AS. Additionally, we identified that CD64 expression on monocytes, both CD14 + CD16 + and CD14 + CD16- subsets, similarly reduces the risk of AS. In terms of inflammatory factors, our findings indicate that elevated levels of TNF-β increase the risk of AS, while, in contrast, lower levels of IL-6 are associated with a decreased risk. Finally, our analysis of serum metabolites revealed that higher levels of hexadecanedioate (C16-DC) and Bilirubin (E, Z or Z, E) are linked to a reduced risk of AS, presenting a nuanced picture of the disease’s pathogenesis through the lens of genetic predisposition and molecular biomarkers. However, the clinical relevance of these findings warrants further scrutiny, given the complexities of translating genetic associations into real-world practice. Our multi-omics MR study identifies potential biomarkers that could guide future diagnostics and therapies for AS, but applying these to improve patient outcomes requires validation through clinical trials. We recognize that our conclusions, based on genetic data, may not fully reflect clinical realities, as MR relies on assumptions like no horizontal pleiotropy, despite our rigorous sensitivity analyses.

In our investigation into the immunopathogenesis of AS, we have identified key immune cell traits markers that delineate the disease’s risk and progression, providing a nuanced understanding of its etiology. The expression of CX3CR1 on monocytes emerges as a critical factor in escalating AS risk, with these cells exhibiting a pro-inflammatory phenotype and an enhanced ability to secrete cytokines such as IL-23 and TNF-like molecule 1A. This cytokine secretion amplifies systemic inflammation and facilitates the interaction with innate lymphoid cell 3, implicating a vital link between gut immunity and systemic inflammatory pathways integral to AS pathogenesis.^[27–29]^ Additionally, the CX3CL1/CX3CR1 axis is implicated in chronic pain and bone resorption, suggesting a broader role in mediating the gut–joint inflammatory axis characteristic of AS.^[29–33]^ Furthermore, the activation and antigen-presenting capabilities of CD62L- CD86+ myeloid DCs are central to orchestrating an immune response that favors AS development. These cells, characterized by their absence of CD62L and presence of CD86, potentiate a Th17-dominated immune response, critical in AS’s inflammatory milieu. This shift towards a Th17 response, coupled with the activated state of these DCs, underscores their significant role in perpetuating the cycle of inflammation characteristic of AS.^[34–36]^ Conversely, the expression of HLA DR on various DC subsets and specific monocyte populations inversely correlates with AS risk, presenting a protective mechanism. HLA DR molecules play a crucial role in antigen presentation to CD4 + T cells, initiating immune responses essential for immune homeostasis. The expression of HLA DR on DCs, myeloid DCs, plasmacytoid DCs, and CD14- CD16- monocytes suggests a regulatory mechanism that mitigates AS risk by promoting immune tolerance, modulating T helper cell responses, and influencing the differentiation pathways of monocytes into DCs with immunoregulatory properties.^[37–39]^ The protective role of CD64 expression on monocytes against AS further complicates the immune landscape. CD64, a high-affinity IgG receptor, is indicative of an activated monocyte state, crucial for phagocytosis and cytokine production. The expression of CD64 on different monocyte subsets (CD14+ CD16+ and CD14+ CD16-) highlights its role in immune surveillance and the initiation of anti-inflammatory responses. Through efficient clearance of pathogens and apoptotic cells, modulation of T cell responses, and regulation of leukocyte trafficking, CD64+ monocytes contribute to reducing AS risk. This comprehensive analysis underscores the complexity of immune cell traits interactions in AS, highlighting the delicate balance between pro-inflammatory and regulatory cell functions. Contrary to its established pro-inflammatory role in Rheumatoid Arthritis, CD64 expression on monocytes correlates with reduced AS risk, a finding that invites deeper investigation into its distinct immunological functions across different inflammatory diseases.^[40]^

In the intricate landscape of AS pathogenesis, our exploration of TNF-β and IL-6 reveals a complex interplay of cytokines that shape the disease’s progression and potential therapeutic avenues. Despite the relative scarcity of research specifically addressing the role of TNF-β in AS, with most studies focusing on TNF-α, our study offers new insights into the relationship. TNF-β, a pivotal pro-inflammatory cytokine, exacerbates AS’s inflammatory milieu by stimulating the production of other cytokines, including IL-1, IL-6, and TNF-α. This amplification of the inflammatory response is crucial for the recruitment and activation of immune cells such as T cells and macrophages, central players in maintaining the chronic inflammation characteristic of AS. The role of TNF-β in the pathogenesis of AS goes beyond simply perpetuating inflammation. Genetic predispositions, such as polymorphisms in the TNF-β gene, can lead to increased expression of this cytokine, thereby heightening susceptibility to AS. This genetic backdrop, coupled with environmental factors, underscores the multifaceted influence of TNF-β in AS’s etiology. Conversely, IL-6 presents a dualistic role within the AS context, traditionally recognized for its pro-inflammatory actions, yet emerging evidence suggests its potential in mitigating AS risk under certain conditions. IL-6 acts at the crossroads of innate and adaptive immunity, influencing the differentiation of T cells into Tregs, pivotal in maintaining immune tolerance and preventing autoimmunity. This induction of Tregs by IL-6, particularly in specific inflammatory or therapeutic contexts, may reduce autoimmune responses against self-antigens in AS, lowering disease risk. Furthermore, IL-6’s involvement in acute inflammation underscores its capacity to limit inflammatory responses, potentially preventing the transition to chronic inflammation, a hallmark of AS pathogenesis.^[41,42]^

The interaction between TNF-β and IL-6 within the AS inflammatory network is nuanced. While TNF-β’s role is primarily pro-inflammatory, driving disease progression by amplifying cytokines like IL-1, IL-6, and TNF-α to sustain chronic inflammation in AS, IL-6 shows a dual role in our findings. We found that lower IL-6 levels are associated with a reduced AS risk (IVW: OR 0.70, 95% CI 0.59–0.85, *P* = .009), suggesting a potential protective effect through mechanisms like Tregs differentiation and immune tolerance under certain conditions. However, this protective association contrasts sharply with IL-6’s well-established pro-inflammatory role in clinical practice for AS. This discrepancy may stem from population-specific genetic variations, unmeasured environmental factors, or context-dependent effects not captured in our GWAS data. Further clinical and mechanistic studies are needed to validate this finding and reconcile it with clinical observations. This dynamic between TNF-β’s pro-inflammatory drive and IL-6’s potential regulatory role highlights opportunities for targeting these cytokines in therapeutic strategies, as modulating their activity with biological agents has shown efficacy in reducing AS inflammation and improving patient outcomes, underscoring the importance of understanding their balance in AS management.^[43,44]^

Building on our research into TNF-related pathways in AS, we also investigated the roles of serum metabolites C16-DC and Bilirubin, uncovering their potential impact on AS’s pathogenesis. Our findings suggest that Bilirubin, traditionally viewed as a byproduct of heme catabolism, possesses antioxidant, anti-inflammatory, and immunomodulatory properties that could offer protection against AS. It effectively neutralizes reactive oxygen species, mitigating oxidative stress and inhibiting critical pro-inflammatory cytokines such as TNF-α, IL-1, and IL-6, which are central to the disease’s inflammatory processes. Furthermore, Bilirubin may influence the IL-23/IL-17 axis, altering the dynamics of immune cells and cytokine profiles involved in AS. Concurrently, hexadecanedioate’s role in fatty acid metabolism points to its influence on mitochondrial function and energy production, both pertinent to chronic inflammatory conditions. By potentially modulating immune cell activation within the IL-23/IL-17 pathway, hexadecanedioate could impact AS’s inflammatory landscape. Its involvement in lipid signaling pathways also suggests a mechanism for exerting anti-inflammatory effects, contributing to a reduced risk of AS.^[45]^

Considering our comprehensive research findings, we propose the immune–metabolic–inflammation network hypothesis as a foundational framework to elucidate the complex pathogenesis of AS. This hypothesis, emerging from an in-depth analysis, suggests that the pathogenesis of AS is significantly influenced by the intricate interplay among immune system dysregulation, metabolic disturbances, and pervasive inflammatory responses. The dynamic interactions between immune cells, metabolites, and inflammatory proteins are identified as critical drivers of AS progression, with each component not only playing distinct roles but also engaging in complex interactions, collectively impacting the disease’s onset, severity, and trajectory.

Central to this conceptual model is the interplay between pro-inflammatory cytokines, such as TNF-β and IL-6, and key immune cells, including monocytes expressing CX3CR1 and myeloid DCs characterized by specific markers. This cytokine-mediated signaling is pivotal, directly impacting immune cell activation, differentiation, and functionality, thereby sustaining the inflammatory milieu intrinsic to AS. Furthermore, the incorporation of serum metabolites like hexadecanedioate and Bilirubin introduces a metabolic dimension that influences immune functionality, with Bilirubin potentially moderating the inflammatory response through its antioxidant and anti-inflammatory properties, and hexadecanedioate affecting immune cell activation and functionality through metabolic pathways. This bidirectional communication between cytokines and metabolites underscores the interdependence of immune responses and metabolic processes, illustrating the necessity of viewing the inflammatory cascade and immune regulation within a broader metabolic context that governs these pathways.

Expanding to a macroscopic perspective, the immune–metabolic–inflammation network offers a systemic lens through which the pathogenesis of AS can be appreciated, highlighting the complex interplay between systemic immunity, metabolism, and inflammation. This perspective transcends individual cellular interactions, focusing on the overarching dynamics that steer disease progression and potential therapeutic interventions. The systemic orchestration of immune responses, mediated by a balance between pro-inflammatory and anti-inflammatory signals, plays a pivotal role in AS’s pathophysiological underpinnings. Additionally, the critical role of metabolism in regulating both systemic immunity and inflammation, influenced by dietary, lifestyle, and environmental factors, directly impacts the immune system’s efficiency and inflammatory response. The interrelation between immune activation and metabolic shifts leads to systemic metabolic alterations, emphasizing the interconnectedness of immune and metabolic processes in AS. Feedback loops between the immune system, metabolic processes, and inflammatory responses are integral to AS progression, highlighting potential avenues for therapeutic intervention.

Our comprehensive analysis, involving 1400 serum metabolites, 731 immune cell traits, and 91 circulating inflammatory proteins, validates the immune–metabolic–inflammation network hypothesis for AS pathogenesis. This holistic framework highlights the intricate interplay between immunity, metabolism, and inflammation as central to AS progression. It underscores the potential of targeted interventions within this network to mitigate inflammation and improve patient outcomes. Emphasizing a unified treatment strategy, our findings advocate for integrated approaches in AS management, pioneering a pathway for future research and therapeutic advancements. This study not only enriches our understanding of AS but also sets a foundation for systemic and multifaceted treatment strategies, marking a significant stride towards innovative AS management.

## 5. Conclusion

The exploration of immune cell traits, serum metabolites, and circulating inflammatory proteins linked to AS offers valuable insights into the pathophysiological mechanisms underlying this condition and unveils promising biomarkers for diagnosis. The proposed hypothesis regarding an immune–metabolic–inflammatory network presents a holistic framework, elucidating the intricate interplay between immune dysregulation, metabolic disorders, and inflammatory responses in the development of AS. These discoveries not only open doors for tailored therapeutic interventions in AS but also present novel pathways for treatment strategies to be explored.

## 6. Limitations

The study faces limitations inherent to MR, including potential bias from horizontal pleiotropy and the use of broad significance thresholds, which may increase the risk of false positives. Our reliance on MR, while robust for causal inference, is limited by its dependence on genetic associations, which may not fully reflect clinical realities due to potential biases like horizontal pleiotropy or unmeasured confounders, despite our sensitivity analyses. Our findings, primarily based on European populations, may lack wider applicability, and the exclusion of environmental and lifestyle factors limits our understanding of AS. Additionally, some findings, such as the protective effect of IL-6 on AS risk, contradict its well-established pro-inflammatory role in clinical practice. This discrepancy may arise from population-specific genetic variations, unmeasured environmental factors, or context-dependent effects not captured in our GWAS data, highlighting a key limitation of our study. Future research should include diverse populations, longitudinal clinical studies, and environmental factors to validate our findings and assess their clinical relevance more comprehensively.

## Acknowledgments

The authors appreciate the summary statistics provided by the original GWAS and related consortia.

## Author contributions

**Conceptualization:** Kai Du, Chen-Yu Zhang, Ao Li, Qi-Heng Zuo.

**Data curation:** Kai Du, Chen-Yu Zhang, Ao Li, Qi-Heng Zuo.

**Formal analysis:** Kai Du, Chen-Yu Zhang, Ao Li, Ren Guo.

**Funding acquisition:** Kai Du, Ao Li, Ren Guo.

**Investigation:** Kai Du, Chen-Yu Zhang, Ao Li, Qi-Heng Zuo.

**Methodology:** Kai Du, Ao Li.

**Project administration:** Kai Du, Chen-Yu Zhang, Ao Li, Ren Guo, Shu-Ming Li.

**Resources:** Kai Du, Chen-Yu Zhang, Ao Li, Qi-Heng Zuo, Shu-Ming Li.

**Software:** Kai Du, Ao Li, Ren Guo, Shu-Ming Li.

**Supervision:** Chen-Yu Zhang, Ao Li, Ren Guo.

**Validation:** Kai Du, Qi-Heng Zuo, Shu-Ming Li.

**Writing – original draft:** Kai Du, Shu-Ming Li.

**Writing – review & editing:** Ren Guo, Shu-Ming Li.

## Supplementary Material


