# Supplementary material for: Unraveling new therapeutic targets in ankylosing spondylitis: Multi-omics Mendelian randomization on immune cells, metabolites, and inflammation proteins
Source: Medicine (Baltimore). 2025 Apr 18;104(16):e42177. doi: 10.1097/MD.0000000000042177 (PMC12014029; doi:10.1097/MD.0000000000042177)

Supplementary Figure 1. Forest Plot of the Association between [CD62L- CD86+ myeloid DC AC] and [AS] using Mendelian Randomization.


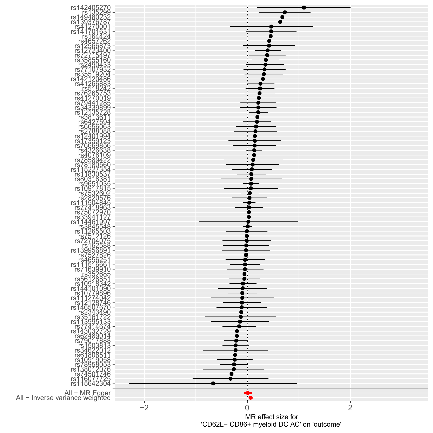


Supplementary Figure 2. Funnel plot of single SNP analysis of [CD62L- CD86+ myeloid DC AC] by Inverse Variance Weighting and MR EGGER on [AS].


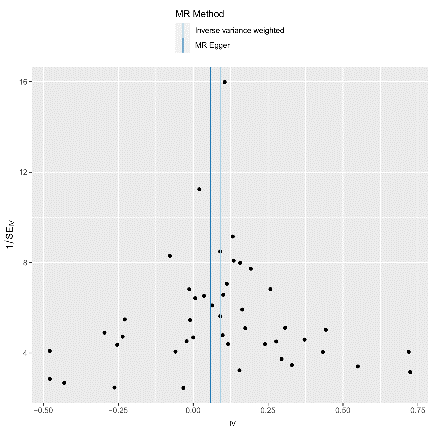


Supplementary Figure 3. Leave-one-out stability tests causal estimates of [CD62L- CD86+ myeloid DC AC] and [AS]


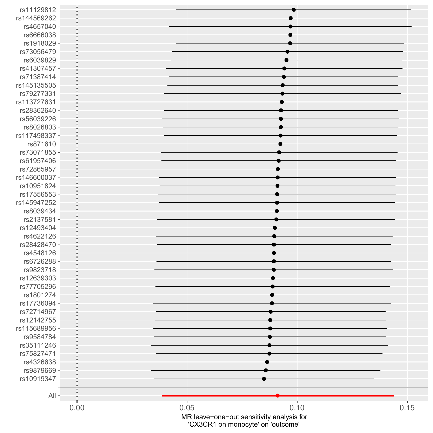


Supplementary Figure 4. Scatter plots of causal estimates of [CD62L- CD86+ myeloid DC AC] on AS. The slope of each line corresponds to the estimated MR effect in different models, including the conventional Inverse Variance Weighted, MR Egger, Simple Mode, Weighted Mode, and Weighted Median.


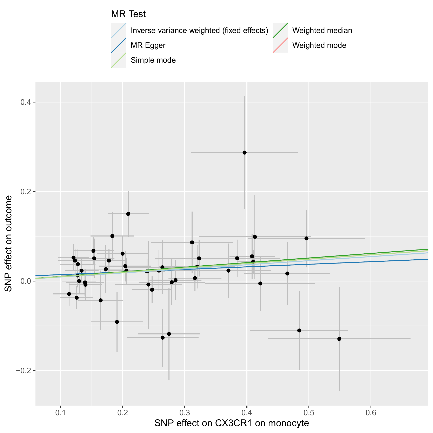


Supplementary Figure 5. Forest Plot of the Association between [CD64 on CD14+ CD16- monocyte] and [AS] using Mendelian Randomization.


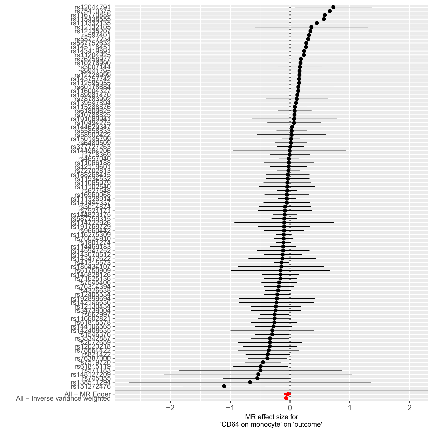


Supplementary Figure 6. Funnel plot of single SNP analysis of [CD64 on CD14+ CD16- monocyte] by Inverse Variance Weighting and MR EGGER on [AS].


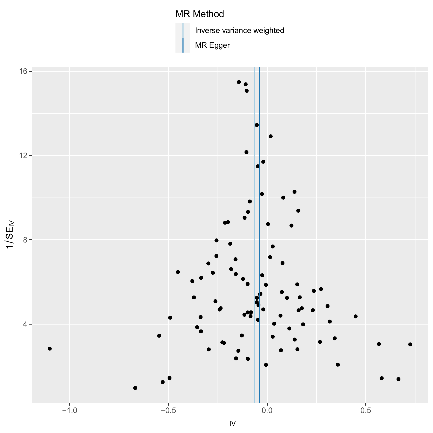


Supplementary Figure 7. Leave-one-out stability tests causal estimates of [CD64 on CD14+ CD16- monocyte] and [AS].


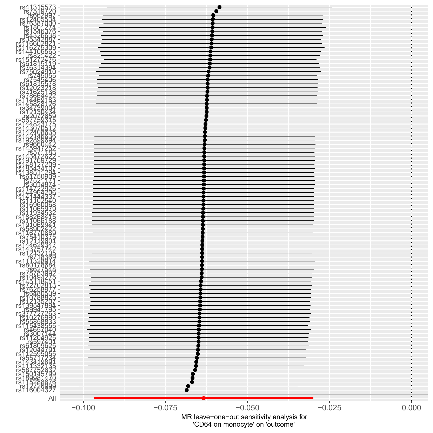


Supplementary Figure 8. Scatter plots of causal estimates of [CD64 on CD14+ CD16- monocyte] on [AS]. The slope of each line corresponds to the estimated MR effect in different models, including the conventional Inverse Variance Weighted, MR Egger, Simple Mode, Weighted Mode, and Weighted Median.


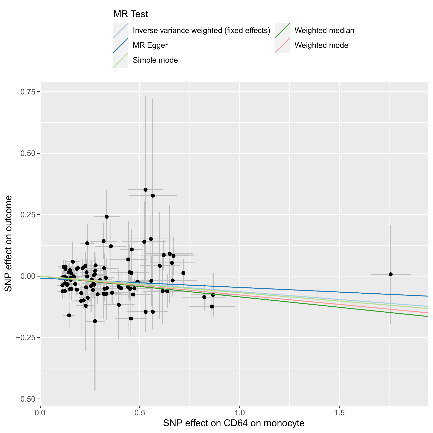


Supplementary Figure 9. Forest Plot of the Association between [CX3CR1 on monocyte] and [AS] using Mendelian Randomization.


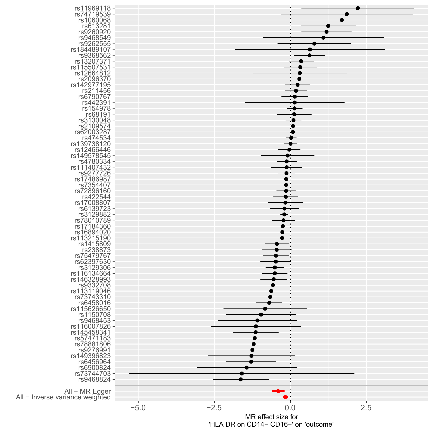


Supplementary Figure 10. Funnel plot of single SNP analysis of [CX3CR1 on monocyte] by Inverse Variance Weighting and MR EGGER on [AS].


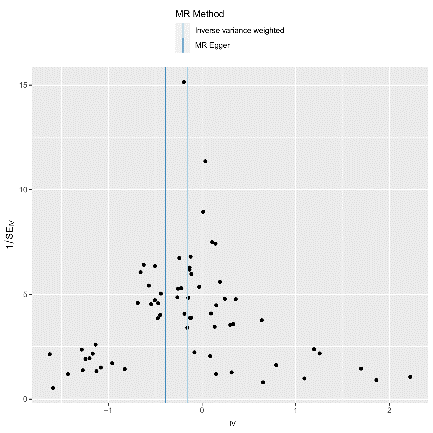


Supplementary Figure 11. Leave-one-out stability tests causal estimates of [CX3CR1 on monocyte] and [AS].


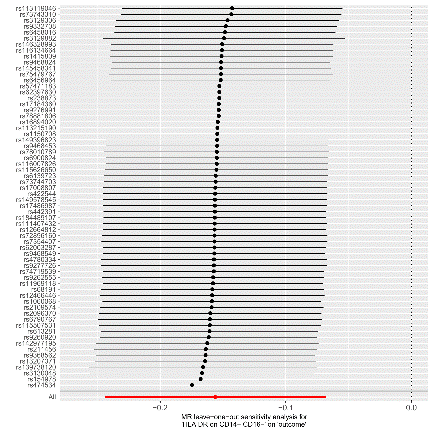


Supplementary Figure 12. Scatter plots of causal estimates of [CX3CR1 on monocyte] on AS. The slope of each line corresponds to the estimated MR effect in different models, including the conventional Inverse Variance Weighted, MR Egger, Simple Mode, Weighted Mode, and Weighted Median.


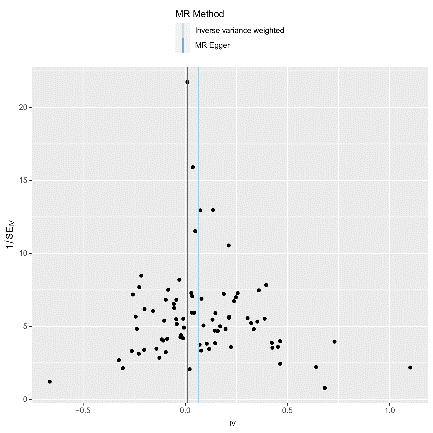


Supplementary Figure 13. Forest Plot of the Association between [CD64 ON MONOCYTE] and [AS] using Mendelian Randomization.


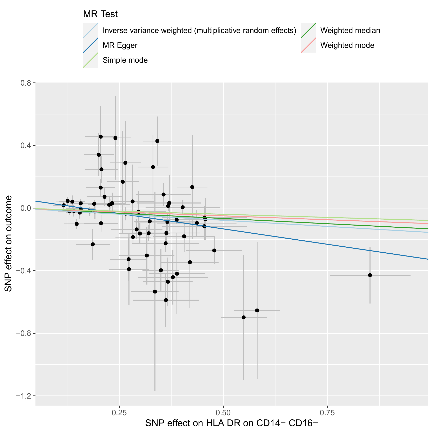


Supplementary Figure 14. Funnel plot of single SNP analysis of [CD64 ON MONOCYTE] by Inverse Variance Weighting and MR EGGER on [AS].


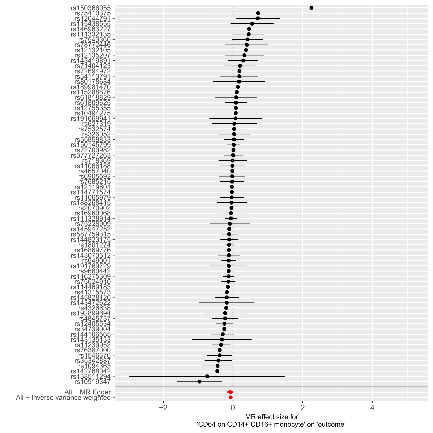


Supplementary Figure 15. Leave-one-out stability tests causal estimates of [CD64 ON MONOCYTE] and [AS].


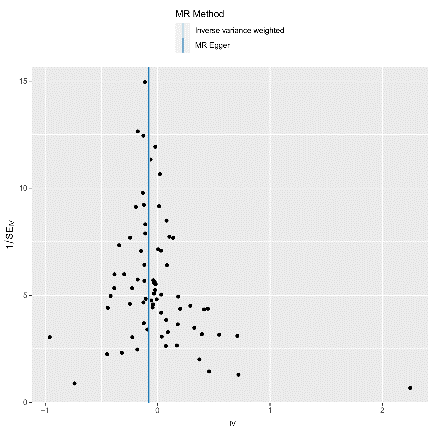


Supplementary Figure 16. Scatter plots of causal estimates of [CD64 ON MONOCYTE] on AS. The slope of each line corresponds to the estimated MR effect in different models, including the conventional Inverse Variance Weighted, MR Egger, Simple Mode, Weighted Mode, and Weighted Median.


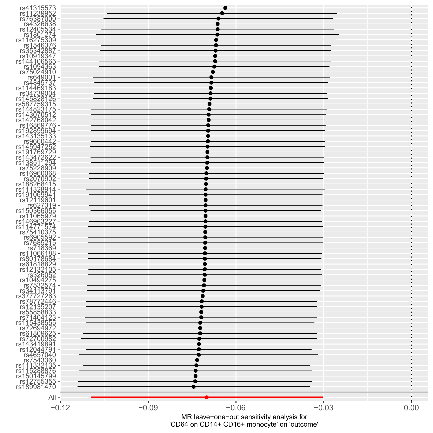


Supplementary Figure 17. Forest Plot of the Association between [HLA DR on CD14- CD16-] and [AS] using Mendelian Randomization.


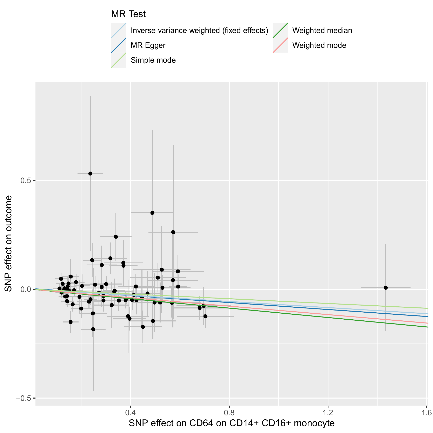


Supplementary Figure 18. Funnel plot of single SNP analysis of [HLA DR on CD14- CD16-] by Inverse Variance Weighting and MR EGGER on [AS].


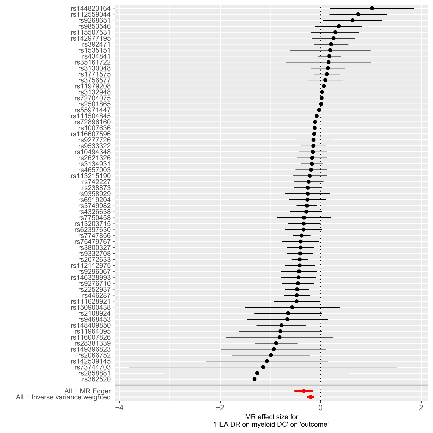


Supplementary Figure 19. Leave-one-out stability tests causal estimates of [HLA DR on CD14- CD16-] and [AS].


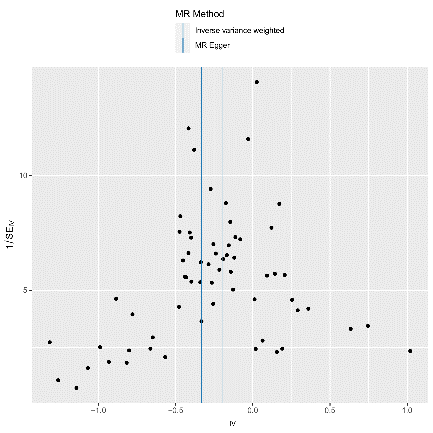


Supplementary Figure 20. Scatter plots of causal estimates of [HLA DR on CD14- CD16-] on AS. The slope of each line corresponds to the estimated MR effect in different models, including the conventional Inverse Variance Weighted, MR Egger, Simple Mode, Weighted Mode, and Weighted Median.


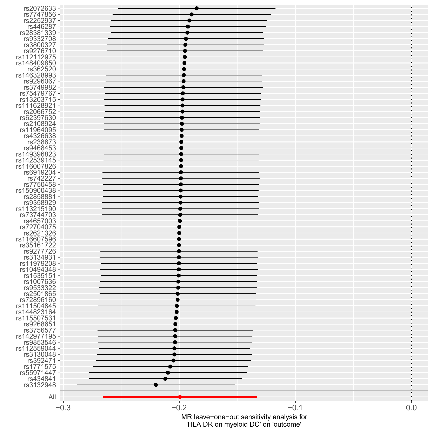


Supplementary Figure 21. Forest Plot of the Association between [CD64 on CD14+ CD16+ monocyte] and [AS] using Mendelian Randomization


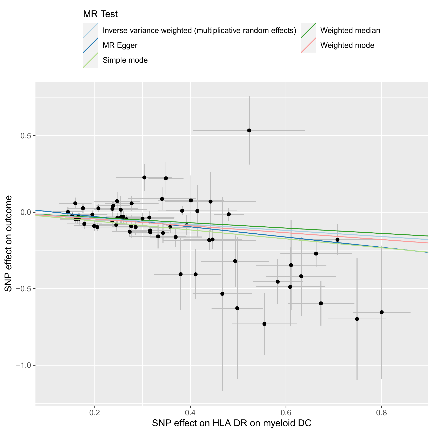


Supplementary Figure 22. Funnel plot of single SNP analysis of [CD64 on CD14+ CD16+ monocyte] by Inverse Variance Weighting and MR EGGER on [AS].


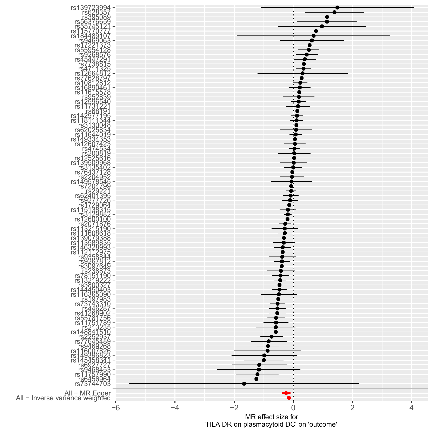


Supplementary Figure 23. Leave-one-out stability tests causal estimates of [CD64 on CD14+ CD16+ monocyte] and [AS].


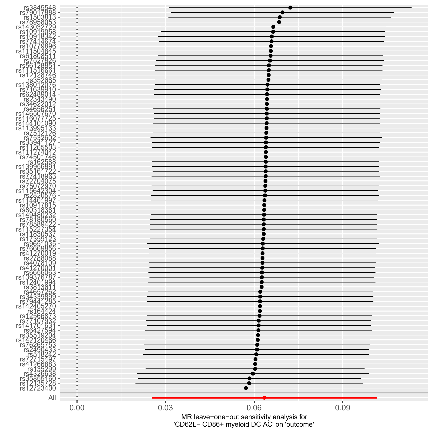


Supplementary Figure 24. Scatter plots of causal estimates of [CD64 on CD14+ CD16+ monocyte] on AS. The slope of each line corresponds to the estimated MR effect in different models, including the conventional Inverse Variance Weighted, MR Egger, Simple Mode, Weighted Mode, and Weighted Median.


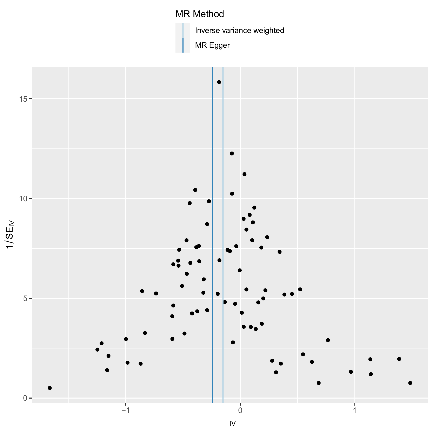


Supplementary Figure 25. Forest Plot of the Association between [HLA DR ON MYELOID DC] and [AS] using Mendelian Randomization.


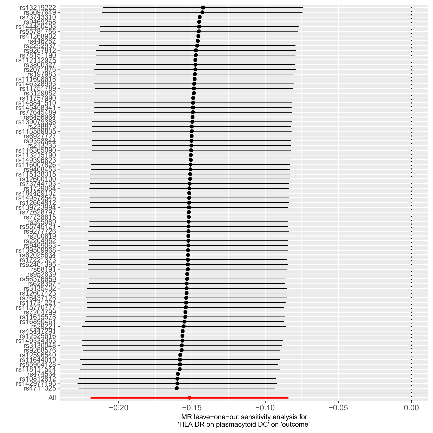


Supplementary Figure 26. Funnel plot of single SNP analysis of [HLA DR ON MYELOID DC] by Inverse Variance Weighting and MR EGGER on [AS].


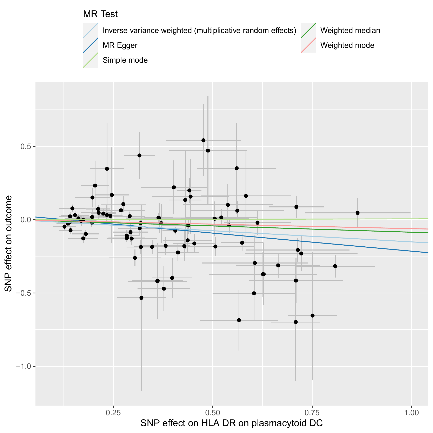


Supplementary Figure 27. Leave-one-out stability tests causal estimates of [HLA DR ON MYELOID DC] and [AS].


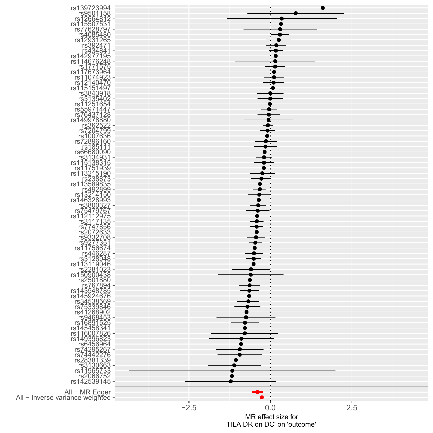


Supplementary Figure 28. Scatter plots of causal estimates of [HLA DR ON MYELOID DC] on AS. The slope of each line corresponds to the estimated MR effect in different models, including the conventional Inverse Variance Weighted, MR Egger, Simple Mode, Weighted Mode, and Weighted Median.


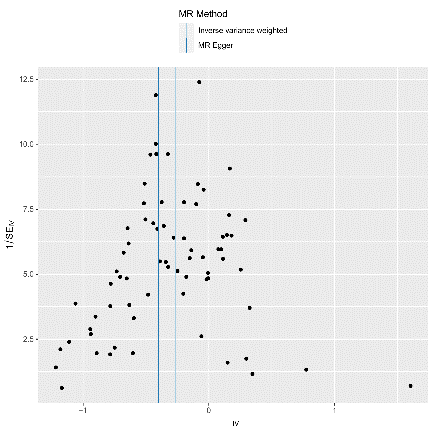


Supplementary Figure 29. Forest Plot of the Association between [HLA DR ON PLASMACYTOID DC] and [AS] using Mendelian Randomization.


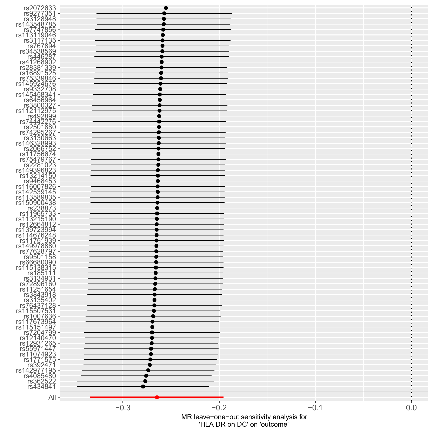


Supplementary Figure 30. Funnel plot of single SNP analysis of [HLA DR ON PLASMACYTOID DC] by Inverse Variance Weighting and MR EGGER on [AS].


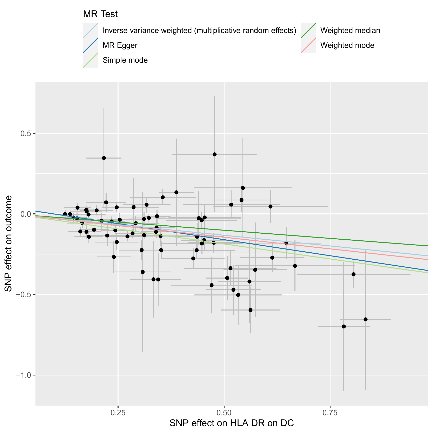


Supplementary Figure 31. Leave-one-out stability tests causal estimates of [HLA DR ON PLASMACYTOID DC] and [AS].


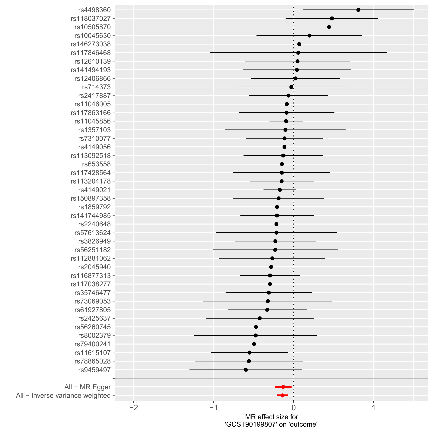


Supplementary Figure 32. Scatter plots of causal estimates of [HLA DR ON PLASMACYTOID DC] on AS. The slope of each line corresponds to the estimated MR effect in different models, including the conventional Inverse Variance Weighted, MR Egger, Simple Mode, Weighted Mode, and Weighted Median.


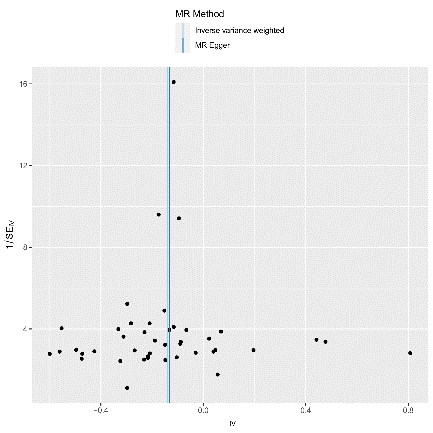


Supplementary Figure 33. Forest Plot of the Association between [HLA DR ON DC] and [AS] using Mendelian Randomization.


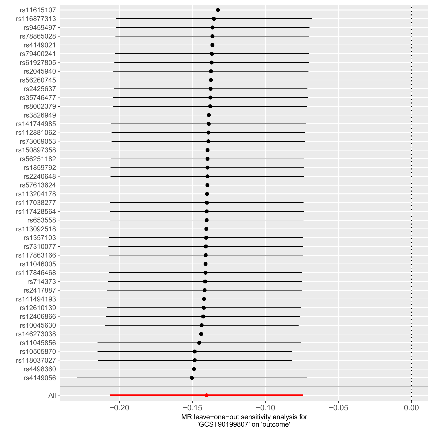


Supplementary Figure 34. Funnel plot of single SNP analysis of [HLA DR ON DC] by Inverse Variance Weighting and MR EGGER on [AS].


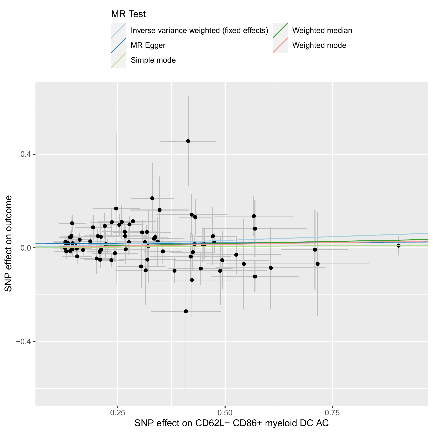


Supplementary Figure 35. Leave-one-out stability tests causal estimates of [HLA DR ON DC] and [AS].


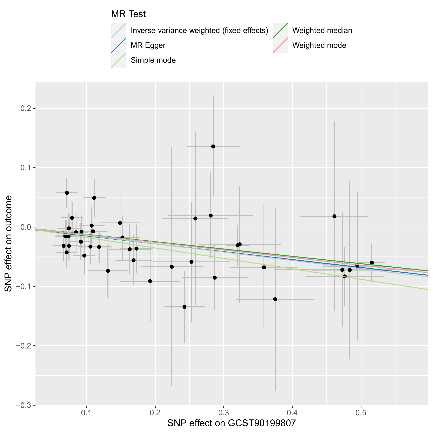


Supplementary Figure 36. Scatter plots of causal estimates of [HLA DR ON DC] on AS. The slope of each line corresponds to the estimated MR effect in different models, including the conventional Inverse Variance Weighted, MR Egger, Simple Mode, Weighted Mode, and Weighted Median.


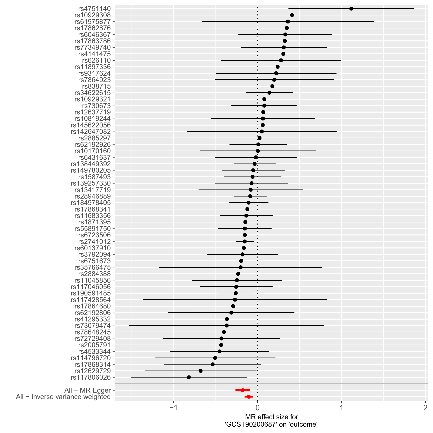


Supplementary Figure 37. Forest Plot of the Association between [HEXADECANEDIOATE (C16-DC) LEVELS] and [AS] using Mendelian Randomization.


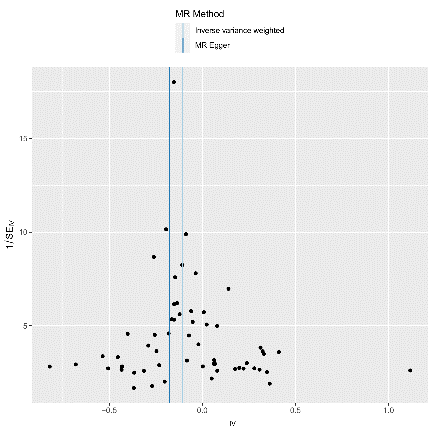


Supplementary Figure 38. Funnel plot of single SNP analysis of [HEXADECANEDIOATE (C16-DC) LEVELS] by Inverse Variance Weighting and MR EGGER on [AS].


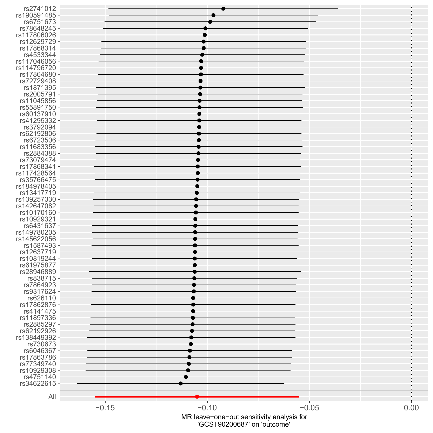


Supplementary Figure 39. Leave-one-out stability tests causal estimates of [HEXADECANEDIOATE (C16-DC) LEVELS] and [AS].


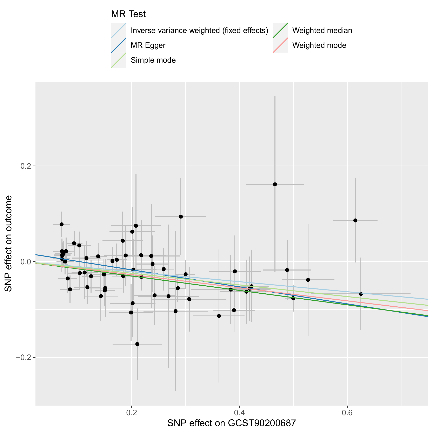


Supplementary Figure 40. Scatter plots of causal estimates of [HEXADECANEDIOATE (C16-DC) LEVELS] on AS. The slope of each line corresponds to the estimated MR effect in different models, including the conventional Inverse Variance Weighted, MR Egger, Simple Mode, Weighted Mode, and Weighted Median.


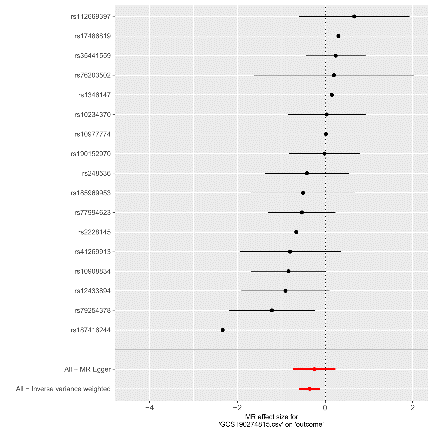


Supplementary Figure 41. Forest Plot of the Association between [BILIRUBIN (E,Z OR Z,E) LEVELS] and [AS] using Mendelian Randomization.


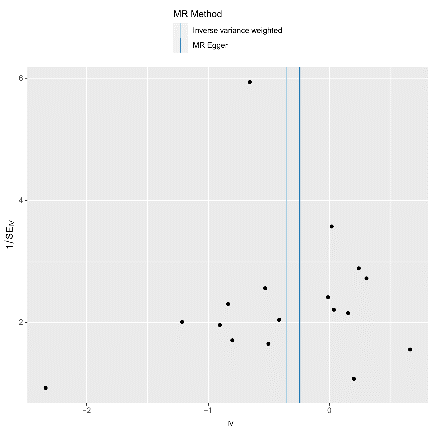


Supplementary Figure 42. Funnel plot of single SNP analysis of [BILIRUBIN (E,Z OR Z,E) LEVELS] by Inverse Variance Weighting and MR EGGER on [AS].


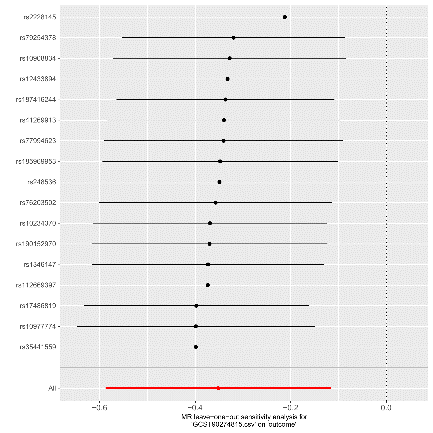


Supplementary Figure 43. Leave-one-out stability tests causal estimates of [BILIRUBIN (E,Z OR Z,E) LEVELS] and [AS].


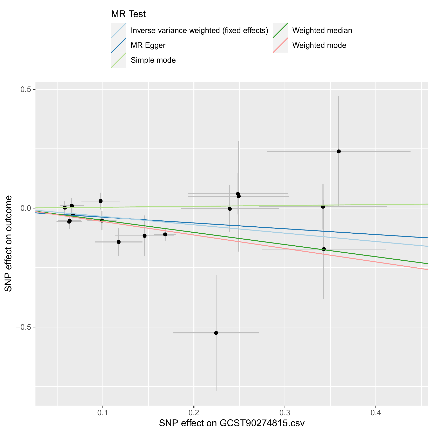


Supplementary Figure 44. Scatter plots of causal estimates of [BILIRUBIN (E,Z OR Z,E) LEVELS] on AS. The slope of each line corresponds to the estimated MR effect in different models, including the conventional Inverse Variance Weighted, MR Egger, Simple Mode, Weighted Mode, and Weighted Median.


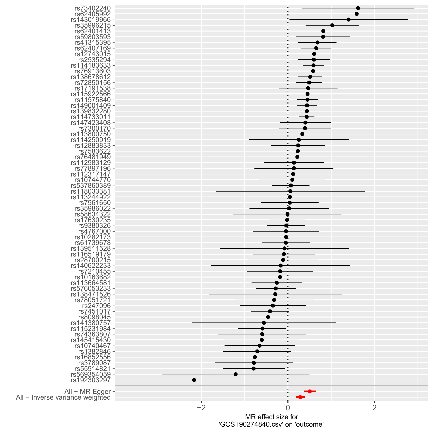


Supplementary Figure 45. Forest Plot of the Association between [INTERLEUKIN-6 LEVELS] and [AS] using Mendelian Randomization.


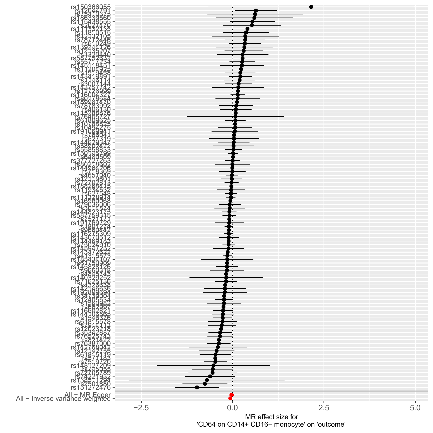


Supplementary Figure 46. Funnel plot of single SNP analysis of [INTERLEUKIN-6 LEVELS] by Inverse Variance Weighting and MR EGGER on [AS].


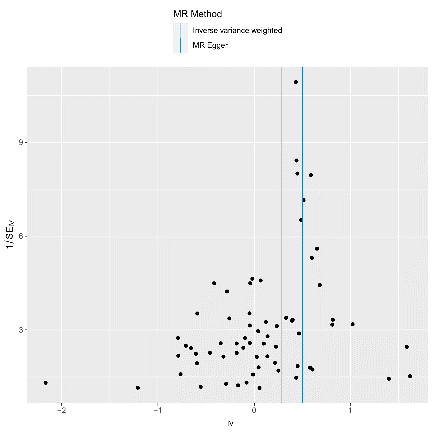


Supplementary Figure 47. Leave-one-out stability tests causal estimates of [INTERLEUKIN-6 LEVELS] and [AS].


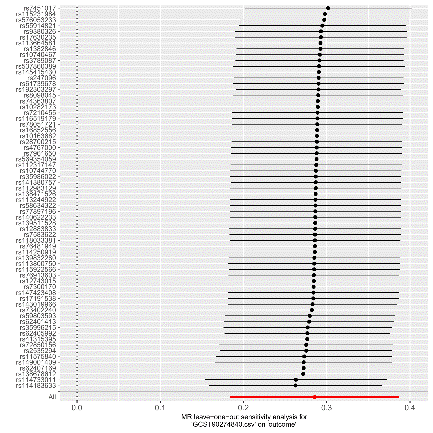


Supplementary Figure 48. Scatter plots of causal estimates of [INTERLEUKIN-6 LEVELS] on AS. The slope of each line corresponds to the estimated MR effect in different models, including the conventional Inverse Variance Weighted, MR Egger, Simple Mode, Weighted Mode, and Weighted Median.


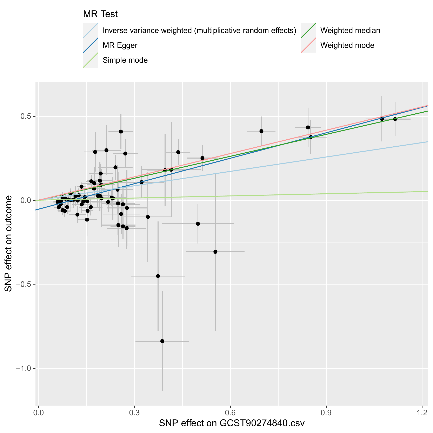


Supplementary Figure 49. Forest Plot of the Association between [TNF-beta levels] and [AS] using Mendelian Randomization.


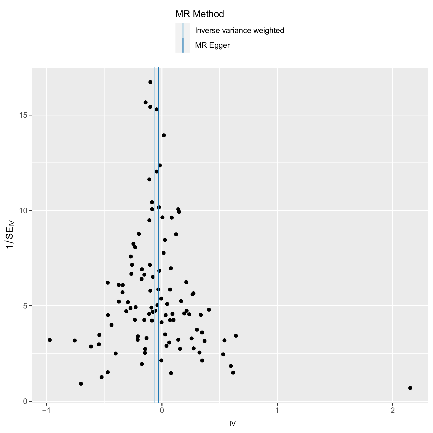


Supplementary Figure 50. Funnel plot of single SNP analysis of [TNF-beta levels] by Inverse Variance Weighting and MR EGGER on [AS].


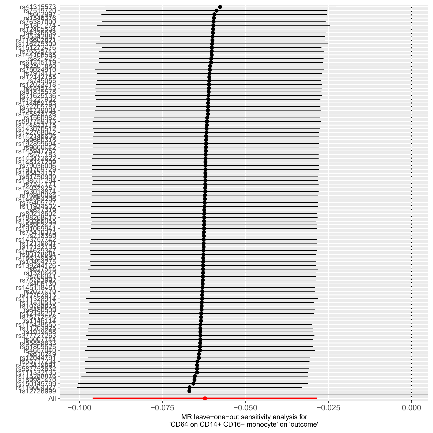


Supplementary Figure 51. Leave-one-out stability tests causal estimates of [TNF-beta levels] and [AS].


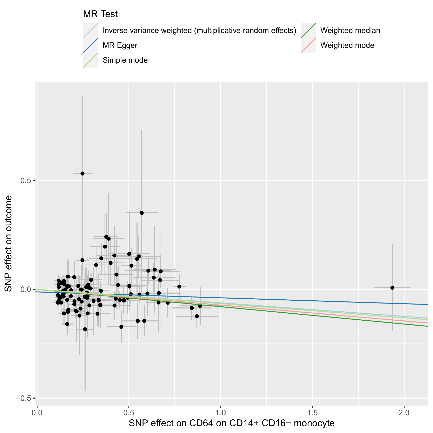


Supplementary Figure 52. Scatter plots of causal estimates of [TNF-beta levels] on AS. The slope of each line corresponds to the estimated MR effect in different models, including the conventional Inverse Variance Weighted, MR Egger, Simple Mode, Weighted Mode, and Weighted Median.


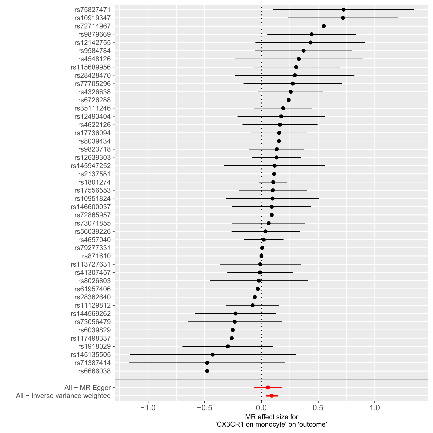

Supplement: Supplementary file 2 [file medi-104-e42177-s002.docx]
